# Health and Well-Being of Older People in the City of Valencia: A Comprehensive Study

**DOI:** 10.3390/healthcare12242526

**Published:** 2024-12-13

**Authors:** Leticia Pérez-Saiz, Mireia Ferri-Sanz, Marina Canas, Mirian Fernandez, Maite Ferrando, Rachael Dix

**Affiliations:** 1 Kveloce (Senior Europa SL), 46003 Valencia, Spain; lperez@kveloce.com (L.P.-S.); mcanas@kveloce.com (M.C.); mferrando@kveloce.com (M.F.); 2 Polibienestar Research Institute, University of Valencia, 46022 Valencia, Spain; mirian.fernandez-salido@uv.es; 3 Valencia Innovation Capital, 46024 Valencia, Spain

**Keywords:** health assessment, older people, value-based healthcare, frailty, well-being, quality of life, lifestyle

## Abstract

Background/Objectives: In the framework of the ValueCare project (funded by EC, ref 875215), the Valencia pilot site assessed the comprehensive health of 240 older people with frailty. ValueCare aims to deliver personalised integrated health and social care and better outcomes for older people. Methods: For the health evaluation, a comprehensive approach was adopted, based on validated questionnaires that address not only mental and physical health but also other key dimensions in older people’s well-being, namely unwanted loneliness and nutrition. Results: This study provides an overview of the overall health status of 60-year-old people from March to December 2022 in the city of Valencia, allowing for a comparison between national and European health indicators. Conclusions: Older people in the city of Valencia reported good health, quality of life, independency, nutrition and social wellbeing, with some differences between gender.

## 1. Introduction

Healthy ageing is defined by the World Health Organisation (WHO) as “the process of developing and maintaining the functional ability that enables well-being in older age [1]”. Healthy ageing involves the adoption of lifestyle and behaviour choices that contribute to overall well-being as people age; personal factors, such as lifestyle and environmental influences, are pivotal [2]. It encompasses the different dimensions of physical, mental, and social health such as physical activity, balanced diet, mental stimulation, social relationships, adequate sleep, and stress management, among others.

It has been widely known that fostering a supportive environment and adopting a healthy lifestyle serve as protective measures against age-related chronic diseases, contributing to the promotion of healthy ageing [3,4]. Despite the longstanding recognition of this general “prescription”, its effective implementation faces challenges arising from both systemic social and environmental barriers to health, particularly in vulnerable populations [5], and individual-level difficulties in sustaining behaviour change over extended periods [6].

Staying healthy involves the capacity to recover to a healthy state after experiencing accidents, illnesses, or stressful events rather than building up compensatory metabolic processes and increasing the detrimental “allostatic load” throughout one’s self [7]. This suggests that an individual’s health “trajectory” can be bidirectional, either moving away from or returning to a state of health as life changes. In contrast, ageing follows a chronologically unidirectional path, and though it cannot be truly “reversed”, it can progress at a variable rate or “pace”, slowing down or speeding up throughout one’s life [8].

In this framework, the ValueCare project (funded by the European Commission, ref 875215) aims at improving the quality of life and health outcomes of older people with cognitive impairment, frailty of multiple chronic diseases, by providing comprehensive and personalised value-based integrated care across Europe. Value-based care represents a contemporary trend in healthcare management [9], defining value as the outcomes significant to patients in relation to associated costs [10]. This approach is closely tied to integrated care delivery, embodying a systemic vision that addresses patient needs while prioritising health and well-being [11]. This paper is focused in assessing the health and well-being status of frailty in older people in the city of Valencia (Spain) where the new intervention model will be tested. According to WHO, frailty is conceptually defined as a clinically recognisable state in which the ability of older people to cope with everyday or acute stressors is compromised by an increased vulnerability brought by age-associated declines in physiological reserve and function across multiple organ systems [12].

In Spain, the prevalence of frailty is 18% according to a recent meta-analysis with people aged over 65 [13]. This prevalence and the current demographic change highlight the importance of frailty in Spain. Moreover, the pandemic aggravated this situation not only because of the effects linked with the COVID-19 pandemic but also due to the measures implemented to reduce the mobility of older people. But positively, frailty is not inherent to the ageing process, it is potentially reversible especially in early stages [13]. Early detection and diagnosis are then essential as it has been demonstrated to be effective.

Consequently, and with the aim to have a deep knowledge of the health and well-being status of the older people in Valencia before implementing the ValueCare model, a comprehensive assessment of the target population’s health and well-being was performed considering patient-reported outcomes measures (PROMs).

## 2. Materials and Methods

**Ethical approval.** Ethical procedures were followed before the recruitment and inclusion of participants in the Valencia study. The study was approved by the Comisión de Ética en Investigación Experimental de la Universitat de València (n° reference, 07-05-2020).

**Recruitment and inclusion criteria**. The recruitment phase targeting frail older individuals in Valencia, Spain, extended beyond eight months. Participants were sourced from seven distinct primary healthcare centres under the jurisdiction of the Valencia City Health Department: República Argentina Health Centre, Alfahuir Health Centre, Salvador Pau Health Centre, Benimaclet Health Centre, Chile Health Centre, and Serrería I Health Centre; all of them belonging to the Malvarrosa-Clinic Health Department. Additionally, to enlarge the scope of the recruitment, one elderly activity centre, namely San José Activity Centre, and two universities catering to older demographics, namely Palacio Ayora and Popular University of Trafalgar, were also included.

Within the healthcare centres, the engagement with health professionals and social workers facilitated the identification of potential candidates based on their familiarity with patients’ medical histories and personal circumstances. At the other centres (universities and activity centre), the initial selection of potential participants was conducted by the researchers through introductory meetings.

Upon the identification of potential participants for the study, researchers checked their compliance with the inclusion criteria (Table A1). The FRAIL scale [14] was employed to evaluate the degree of **frailty** among participants. This widely used tool offers a succinct and rapid assessment method, aiding in the identification of frailty based on five primary components: fatigue, resistance, ambulation, illness, and loss of weight. A score of ≥1 on this scale indicates a high likelihood of frailty. The dependency level was assessed by using the Barthel index [15]. This ordinal scale evaluates an individual’s capability to perform ten activities of daily living (**ADLs**), providing a quantitative measure of their functional capacity in these areas. Activities encompassed within the index include eating, personal grooming, toileting, bathing/showering, transferring between seated and lying positions, mobility (walking or wheelchair use), stair navigation, dressing, and continence control. Scores exceeding 60 points on this index indicate a high level of dependency. Finally, a **cognitive impairment** assessment was performed employing the short portable mental status questionnaire (SPMSQ) [16]. Comprising ten questions, this brief assessment tool explores various cognitive domains, including orientation, memory recall, concentration, and calculation abilities. A score falling within the range of 0–2 on this questionnaire is indicative of significant cognitive impairment.

**Inform consent**. Participants who met the described inclusion criteria were provided with a detailed informed consent document to read and sign prior to their involvement in this study. Informed consent was characterised by voluntariness and absence of coercion, predicated on a comprehensive comprehension of the research’s objectives, procedures, and the nature of personal data processing involved. Furthermore, the document stipulated that participant retained the prerogative to discontinue their participation in the study at any juncture.

**Data collection.** Data were gathered through self-administered questionnaires completed by older individuals, with assistance provided by the research team when needed. The questionnaire was based on patient-reported outcomes measures (PROMs); concretely, some of the instruments included in the standard set for older person established by the International Consortium for Health Outcomes Measurement (ICHOM dataset) [17,18] and extra-validated questionnaires to complete the health and well-being assessment of older people with two key domains: nutrition and physical performance. Specific details of the instruments are detailed in Table A2. It is important to note that the authors used validated translations when available. When they were not available, the translation was conducted using the back-translation method to ensure cultural adaptation and linguistic accuracy. Moreover, before starting the study, the entire questionnaire was tested with end-users to ensure its user-friendliness in terms of appropriateness, comprehensibility, and usability. Once data were collected, researchers transcribed the paper-based data into the Generic Medical Survey Tracker (GemsTracker) software (https://esurvey.erasmusmc.nl/valuecare/ask, accessed on 1 December 2024), chosen for its robust security features and its capacity for data collection, submission, and modification.

### Outcome Measures Used in the Comprehensive Evaluation of Health

The outcomes of interest and the instruments used to measure them are described in Table A2. First, the authors focused on the **health-related quality of life** (HR-QoL) score, measured via the application of the patient-reported outcomes measurement information system—global health (PROMIS-10) [19] and the EuroQol-5 dimensions (EQ5D5L) [20]. The PROMIS-10 comprises a 10-item survey designed to capture dimensions encompassing physical health, mental well-being, satisfaction with social engagements, and overall quality of life. Renowned for its validation through contemporary psychometric techniques and computerised adaptive testing methodologies, the PROMIS-10 ensures enhanced precision and diminished respondent burden in measurement. Additionally, by using EQ5D5L, the authors evaluated the generic health status. This standardised instrument is widely used in HR-QoL assessment based on the following five dimensions: the ability to walk and move around (mobility); the ability to wash and dress oneself (selfcare); the ability to perform usual activities such as housework, family, or leisure activities (usual-activities); the presence and severity of pain or discomfort (pain/discomfort); and the presence and severity of anxiety or depression (anxiety or depression).

Other important outcome measures encompassed a spectrum of **health and well-being** indicators, spanning physical and psychological frailty, comorbidities, loneliness, functional capacity in activities of daily living, and falls. For measuring **frailty**, the Tilburg frail indicator was used [21]. **Comorbidities** were measured using the ICHOM older person set. The University of California, UCLA, 3-item scale [22] assessed the following three dimensions of **loneliness** among older people: relation connectedness, social connectedness, and self-perceived isolation. Finally, the ability to perform **activities of daily life** was assessed using the modified 10-item Barthel index [23], which informs about the functional status of respondents. Other outcome measures as well as **lifestyle behaviours** concerning smoking and alcohol consumption were also assessed through the ICHOM older person set. Moreover, physical activity was measured using a combination of the SHARE-frailty [24] and the international physical activity, IPAC, questionnaire [25]. Nutrition measures were determined by the short nutrition assessment questionnaire SNAQ65+ [26], which measures different concepts of malnutrition and therefore identify different persons at risk; and for the medication intake, we used the medication risk questionnaire (MRQ-10) [27].

Finally, the **care use** was evaluated through the modified self-management resource centre (SMRC) Healthcare utilisation questionnaire [28], which provides information about the frequency and duration of hospital admissions.

These methods and instruments were chosen for their patient-centred nature, extensive validation, and capacity for self-administration.

- **Sociodemographic data of the sample.** A total of 242 participants (74.79% women) (average age = 73.8; standard deviation of 14.7) were included in the study. Table A3 provides an overview of the sample sociodemographic data.

**Comprehensive health assessment. Data analysis.** Statistical analysis was carried out using GraphPad Prism6TM. Statistical comparison between two populations was performed using unpaired two-tailed Student’s t-test or Mann–Whitney U-test non-parametric two-tailed test when the data failed either the Kolmogorov–Smirnov or a Shapiro–Wilk normality tests. Gender group comparison was performed using *t* test. *p* values < 0.05 were considered statistically significant and set as follow: * *p* < 0.05; ** *p* < 0.01 and *** *p* < 0.001.

## 3. Results

### 3.1. Health, Well-Being and Quality of Life

**Figure A1:**
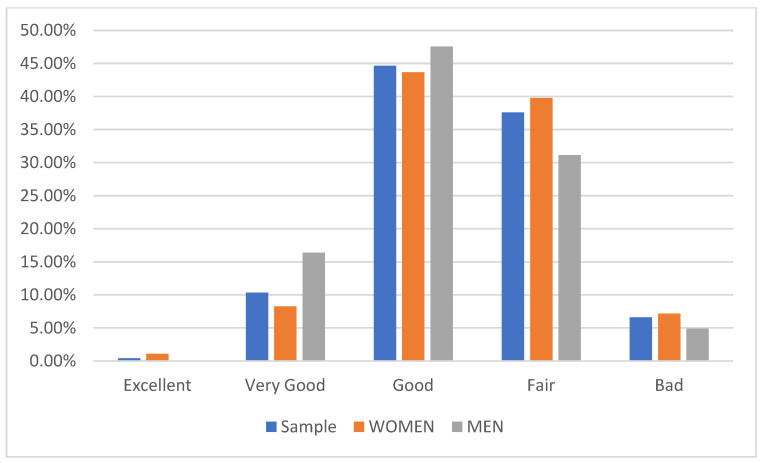
General Health, physical health, and mental health by gender.

**Health.** The assessment of overall health status was conducted using the PROMIS-10 instrument. Considering the health dimensions breakdown, a substantial proportion of the sample self-reported their health status as either good (44.63%) or fair (37.6%). A minority of respondents described their health as “very good” (10.33%), with a notably smaller fraction (0.4%) attributing the descriptor “excellent” to their health. Conversely, a small segment of the population (6.6%) reported their health as “bad,” while no individuals characterised their health as “very bad.” Considering gender, men and women reported the same physical health, but men reported a high score in the mental health. In the different items, men reported better general health than women (see Figure A1), although not many men reported an excellent health status.

T-score distributions of PROMIS health are standardised such that 50 represents the average (mean) for the US general population, and the standard deviation around that mean is 10 points. In our research, concerning the **PROMIS physical health T score**, 22 individuals achieved scores surpassing 50. The mean score observed was 42.6, with a standard deviation of 4.34. Conversely, regarding the **PROMIS mental health T score**, 185 participants obtained scores exceeding 50. The mean score recorded for mental health was 44.8, accompanied by a standard deviation of 3.67. The average physical and mental health of the study participants were not significantly different compared with the general population mean. (*p*-value for physical health = 0.3850 and *p*-value for mental health = 0.4572).

Comparing the means of T score for men and women vs. the mean of the general population, no significant differences were found regarding neither physical nor mental health. However, comparing men vs. women within participants, men showed improved physical (T score 45.7 for men vs. 41.53 for women; t = 3.452; *p*-value *** = 0.0007) and mental health (T score 47.9 for men vs. 43.76 for women; t = 4.154; *p*-value < 0.0001) scores compared to women.

**Well-being and quality of life**. The EQ5D5L score refers to a standardised measure used to assess health-related quality of life. It evaluates the following five dimensions of health: mobility, self care, usual activities, pain/discomfort, and anxiety/depression, providing a comprehensive overview of an individual’s health status. Higher scores indicate better health-related quality of life, while lower scores suggest poorer health status. The EQ5D5L score for the general population was 0.75 (with 1 representing the optimal health state). Although the score of the study participants is lower than 1, the differences were not significant. Considering gender, the score was higher for men (0.87) than for women (0.70), indicating better quality of life for the former (t = 4.436, *p*-value *** < 0.0001).

**Figure A2:**
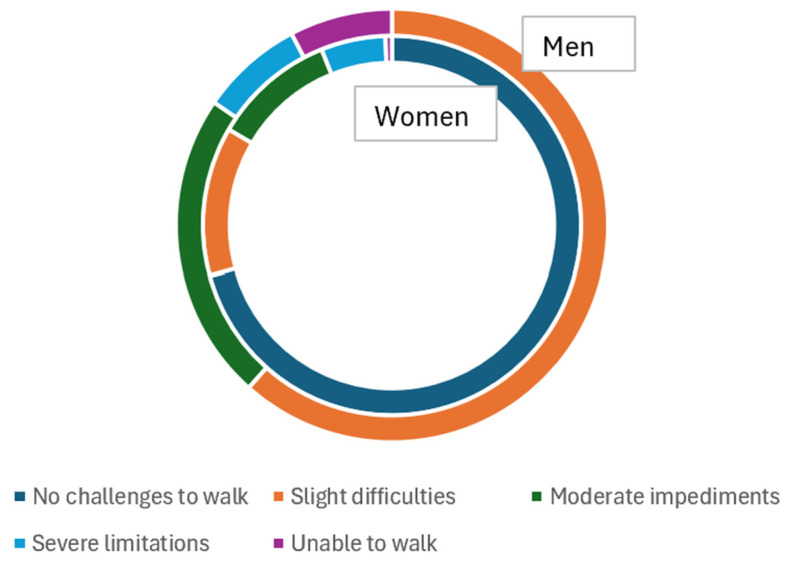
Mobility by gender.

**Mobility**. The mobility index for the sample was 1.48 (1.52 for women and 1.34 for men). Of the total participants, 176 individuals (72.8%) reported no challenges to walk, while 31 individuals (12.8%) experienced slight difficulties, 22 individuals (9.09%) faced moderate impediments, and 11 individuals (4.55%) encountered severe limitations. Only two (0.83%) individuals were unable to walk. In general, women reported better mobility than men, as detailed in the Figure A2. However, these differences were not significant (t = 1.361; *p*-value = 0.1794).

**Figure A3:**
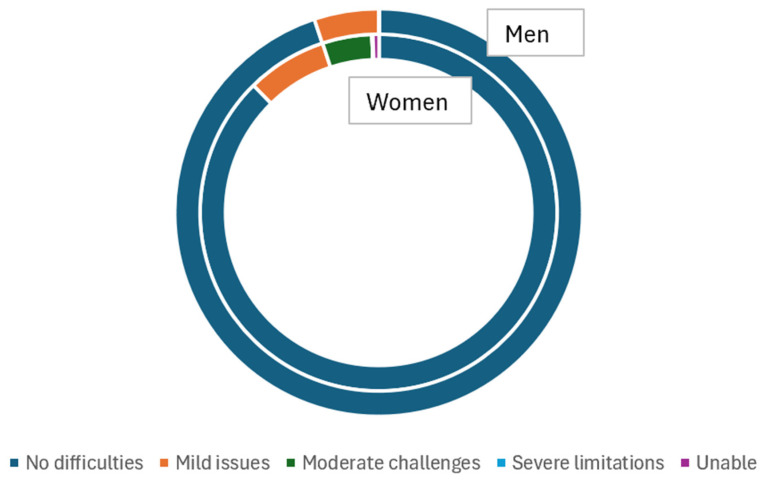
Selfcare by gender.

**Selfcare**. Out of the entire sample, 212 individuals (87.6%) indicated no difficulties with personal hygiene tasks such as washing or dressing. Conversely, sixteen individuals (6.61%) reported mild issues, ten individuals (4.13%) experienced moderate challenges, three (1.24%) individuals encountered severe limitations, and only one (0.41%) individual was unable to perform these activities. Selfcare index was 1.2 for the sample. Again, men reported less issues regarding selfcare tasks than women (selfcare index for men 1.11 vs. 1.23 for women), as described in Figure A3. However, again these differences were not significant (t = 1.305; *p*-value = 0.1931).

**Figure A4:**
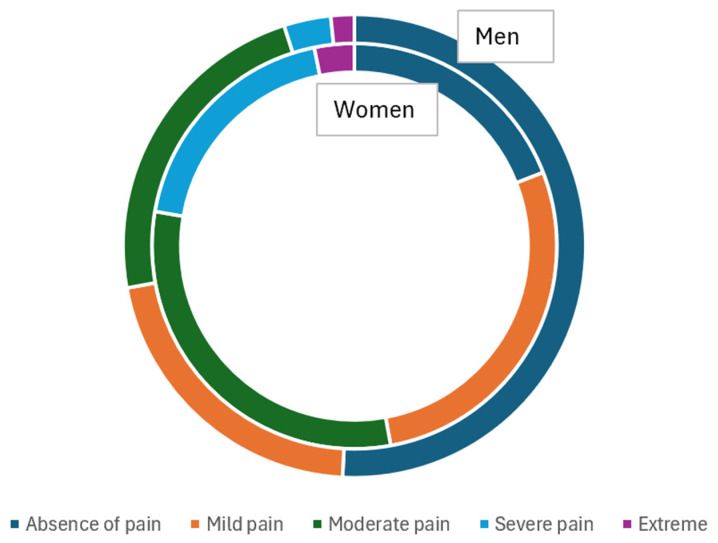
Pain by gender.

**Pain**. Of the total cohort, 85 individuals (35.12%) reported absence of pain or discomfort, while 57 individuals (23.55%) experienced mild pain. Additionally, sixty-two individuals (25.62%) reported moderate pain, thirty-two individuals (13.22%) reported severe pain, and six individuals (2.48%) reported extreme pain. Sample pain index was 2.24. Regarding sex-segregated data analysis, it was observed that male respondents indicated comparatively significant lower levels of pain when contrasted with their female counterparts (men pain index 1.83 vs. women pain index: 2.38; t = 3.288; *p*-value ** = 0.0012) as can be observed in Figure A4.

**Figure A5:**
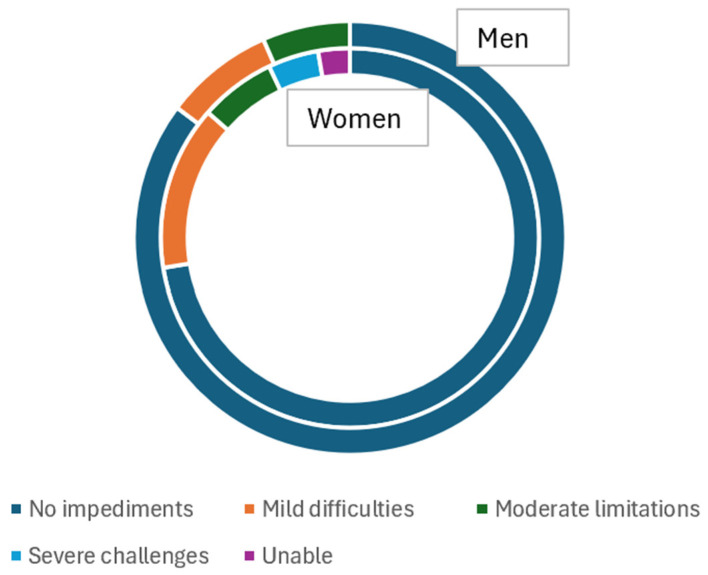
Daily activities by gender.

**Daily activities**. Among participants, 186 individuals (76.6% of the population) experienced no impediments in executing their daily tasks. Conversely, sixteen individuals (6.61%) reported mild difficulties, ten individuals (4.13%) experienced moderate limitations, three individuals (1.24%) faced severe challenges, and only one (0.42%) individual was incapable of performing daily activities. Daily activity index was 1.38. While most individuals in both cohorts reported no difficulties in performing their daily activities, there were instances among women where some indicated limitations in their ability to carry out these tasks, with a subset reporting an inability to do them. Conversely, no instances were reported among men wherein severe challenges or an inability to perform daily activities were noted, see Figure A5. Difference in the capacity of performing daily activities between women and men were not significant (daily activity index for men 1.1 vs. daily activity index for women 1.43; t = 1.882; *p*-value = 0.0610).

**Figure A6:**
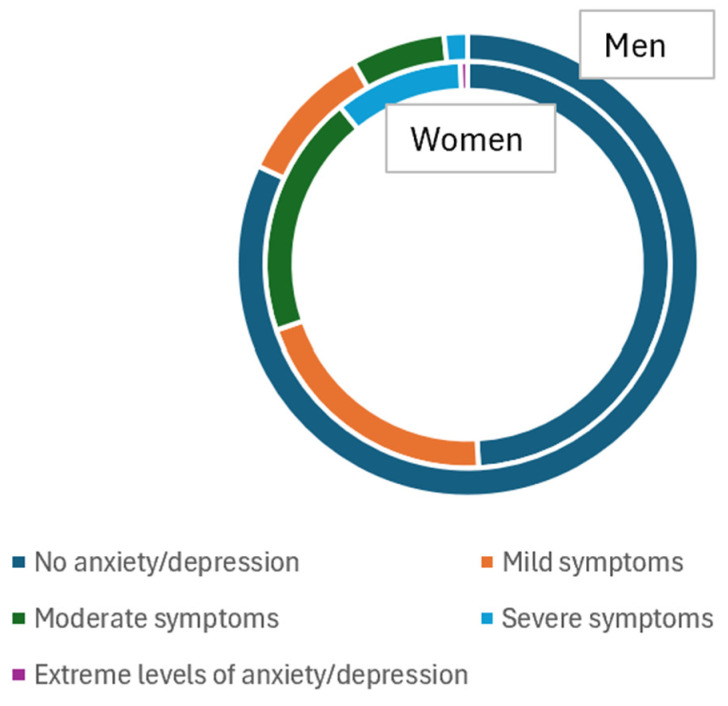
Anxiety/depression by gender.

**Anxiety/depression.** Of the total sample, 136 individuals (56.2%) reported neither anxiety nor depression. However, forty-two individuals (17.36%) reported mild symptoms, thirty-eight individuals (15.70%) experienced moderate symptoms, nineteen individuals (7.9%) reported severe symptoms, and seven individuals exhibited extreme levels of anxiety or depression. Anxiety/depression index was 1.84. When considering gender-based comparisons (see Figure A6), it was observed that a predominant proportion of male participants reported an absence of symptoms associated with anxiety or depression. Conversely, less than half of the female participants reported similar symptomatology, with instances of mild, moderate, and severe manifestations documented (refer to Figure A6). Consequently, the prevalence of anxiety/depression symptoms among women significantly exceeded that among men (anxiety/depression index for women: 2.03 vs. anxiety/depression index for men 1.27; t = 4.674; *p*-value *** < 0.0001).

**Frailty.** The Tilburg frailty indicator (TFI) was employed as the assessment tool to evaluate frailty within our target group. On a continuum ranging from zero to fifteen, individuals with a TFI equal to or exceeding five are classified as frail. Within our study population, 100 individuals (41–32% of the population) were identified as frail (TFI ≥ 5), while 142 individuals (58.68% of the population) were categorised as non-frail (TFI < 5). The TFI for the general population was 5.62. Regarding **physical frailty** (TFI = 0–8), 114 participants (47.11%) were identified as physically frail (TFI = 3–8), while 126 individuals (52.07%) exhibited no signs of physical frailty (TFI = 0–2). The TFI for the general population was 2.90. In terms of **psychological frailty** (TFI = 0–4), 114 individuals manifested psychological frailty (TFI = 2–4), whereas 126 participants did not exhibit psychological frailty (TFI = 0–1). The TFI for the general population was 1.50. Lastly, concerning **social frailty** (TFI = 0–3), 92 individuals were deemed to have social frailty (TFI = 2–3), while 148 individuals did not display social frailty (TFI = 0–1). The TFI for the general population was 0.95.

**Figure A7:**
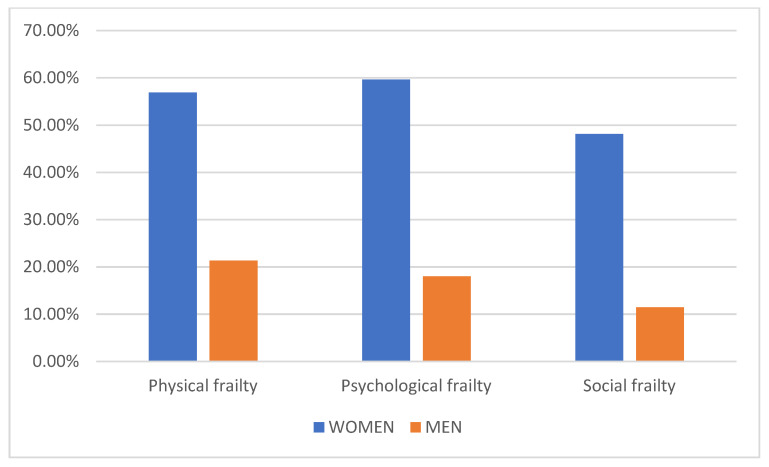
Frailty by gender.

Comparing frailty between women and men, we found that women presented increased levels of frailty than men (total TFI for women 5.92 and for men 5.31) with *p*-value *** < 0.0001 (t = 5.393). As depicted in Figure A7, the prevalence rates of physical frailty, psychological and social frailty are notably elevated among women compared to men. Specifically, for the former two categories, more than half of the female participants perceive themselves as physical frail, with higher TFIs than men (TFI physical score for women was 3 and for men was 2.8; t = 2.464; *p*-values *** < 0.0001), while nearly half perceive themselves as socially frail, also showing higher TFIs than men (TFI psychological score for women was 1.6 and for men 0.7; t = 5.043; *p*-value * = 0.0144).

**Figure A8:**
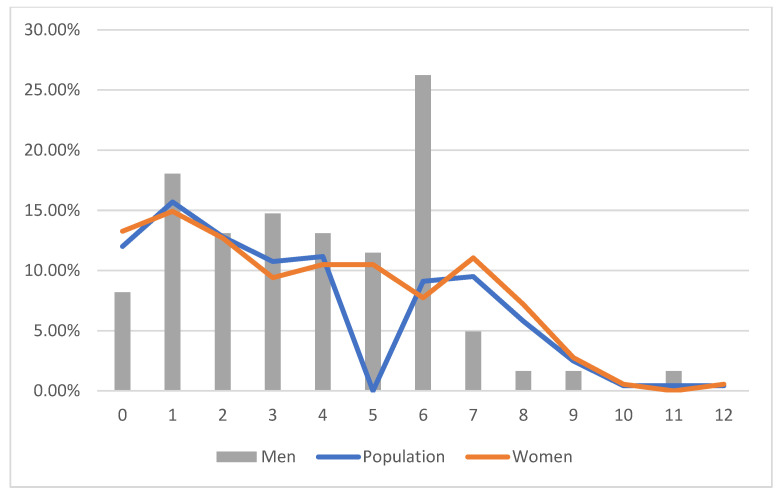
Comorbidities.

- **Loneliness.** The UCLA loneliness scale served as the instrument for assessing loneliness within the study cohort. Most participants (88%, 213 individuals) reported absence of loneliness, as indicated by UCLA scores ranging between three and five. Conversely, 29 individuals (12%) acknowledged experiencing feelings of loneliness, as evidenced by UCLA scores falling within the range of six to nine. Considering gender, 14.92% of women feel lonely, while this percentage for men is 3.28%. Moreover, when comparing the UCLA score between men (3.336) and women (3.973), the difference was significant (t = 3.633; *p*-value*** = 0.0003);
- **Comorbidities.** The number of comorbidities for older people in the city of Valencia assessed through the ICHOM older person set is summarised in Figure A8. The mean comorbidities for older people in the city of Valencia is 3.7. This value is similar for men (3.52) and women (3.77), without significance differences (t = 0.64; *p*-value = 0.5226).

### 3.2. Physical Functioning

**Figure A9:**
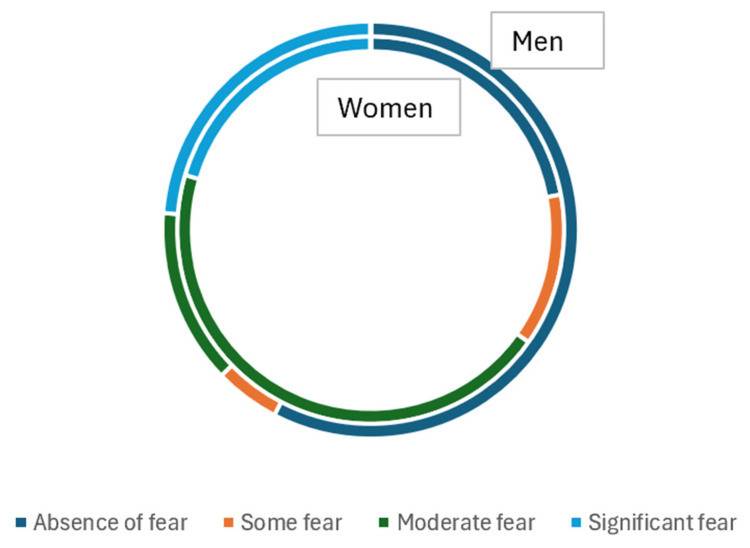
Falls data by gender.

- **Dependency level.** The level of dependency was assessed through the Barthel instrument, to assess functional capacity in basic activities of daily living. Activities of daily living (ADLs) such as controlling bowels, bathing, dressing, feeding, using the toilet, transferring, and mobility were evaluated. For the ADLs, we found that the vast majority of the population was at low risk based on the Barthel ADL score (238 individuals, 98.35%), whereas only 4 individuals (1.65%) were at moderate risk of dependence. For mobility, the Barthel mobility score revealed 229 individuals (94.63% of population) at low risk of dependence and 13 individuals (5.37%) at moderate risk. Values are similar for women and men and differences are not significant (Barthel ADL for women was 98.35% and for men 98.36%; *p*-value for ADL = 0.4076; Barthel mobility for women was 94.48% and for men 95.08%; t = 0.8295; *p*-value for mobility = 0.2952);
- **Falls.** Approximately 30.6% of the study population reported experiencing at least one fall within the preceding year. Conversely, the remaining 169 participants did not recall experiencing any falls during the same period. In relation to **fear of falling (FoF),** as assessed on a scale ranging from one to ten, findings revealed that 30.6% of the participants (74 individuals) indicated an absence of fear of falling (scoring zero on the scale). Furthermore, 10.7% of the population (26 individuals) reported experiencing “some fear” (scoring between two and three on the scale), while 21.1% (51 individuals) acknowledged experiencing fear of falling to a moderate extent (scoring between three and five on the scale). Additionally, 36.8% of the participants (89 individuals) admitted to experiencing a significant degree of fear of falling;
- Overall, the data indicates that men experience slightly fewer falls compared to women, with a lower incidence of falls reported in the past year (see Figure A9). Additionally, in terms of fear of falling, women appear to exhibit higher levels of apprehension; A total of 65.19% of women express moderate or significant fear, whereas more than half of the men report no fear of falling. When we compared the fear of falling score between women (4.689) and men (2.098), we found significant differences (t = 5.210; *p*-value **** < 0.0001).

### 3.3. Lyfestyle

**Figure A10:**
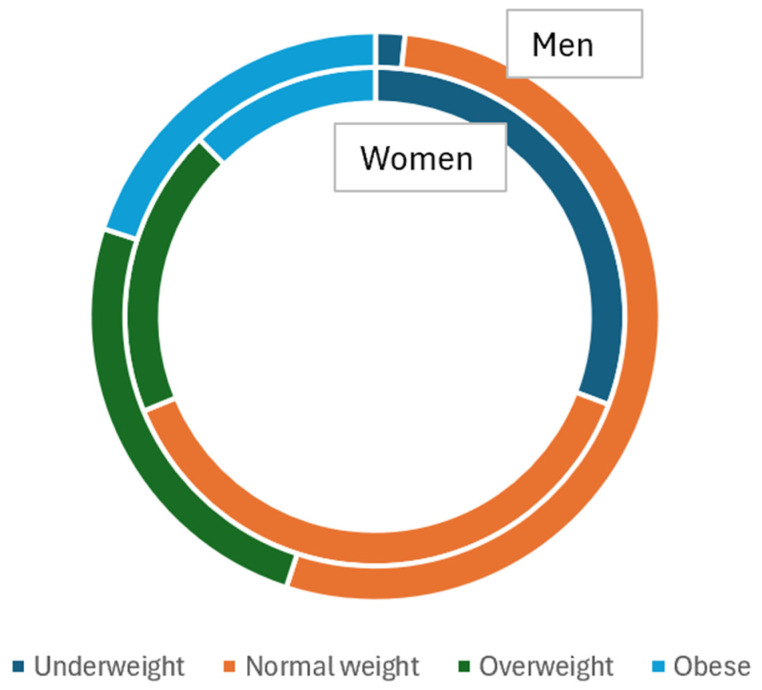
BMI by gender.

**Figure A11:**
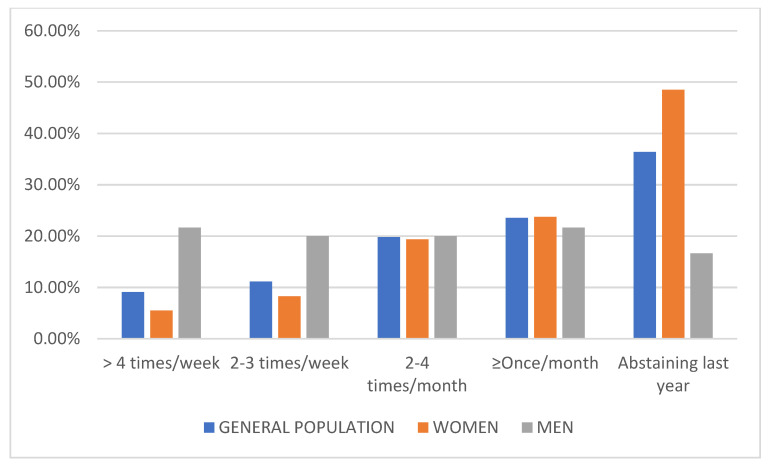
Alcohol consumption by gender.

**Figure A12:**
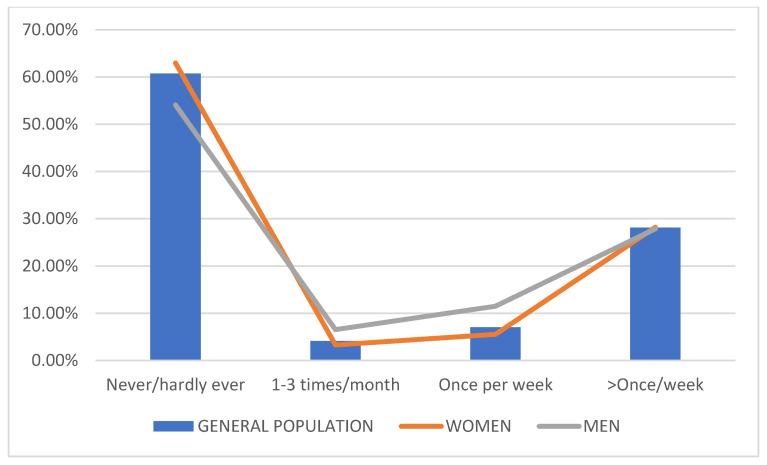
Physical activity.

- **Body mass index (BMI)** of each participant was measured using their respective weight and height measurements. Our analysis revealed that among the participants, 31 individuals (constituting 12.8% of the sample) were classified as underweight, while 115 individuals (47.52%) fell within the normal weight range. Furthermore, 57 participants (23.55%) were categorised as overweight, and 39 individuals (17.12%) were identified as obese. These findings indicate that less than half of the surveyed population exhibits a normal weight status. Specifically, it is noteworthy that approximately 00000.67% of the population is characterised as overweight or obese. Data by gender are presented in Figure A10. Comparisons between the averages for the BMI for women and men showed significant differences, indicating a higher BMI for men (mean BMI for women was 26,33 and for men 27.95; t = 2.629; *p*-value ** = 0.0091);
- **Smoking.** Data indicate that out of the total population surveyed, there were 25 individuals (10.33%) who currently smoke, 82 individuals (33.88%) who were former smokers, and 135 individuals (55.79) who have never smoked. Consequently, it is evident that most of the population, comprising almost 90% (89.67), is presently not engaged in smoking behaviour. When examining these data based on gender, it is evident that the prevalence of smoking is slightly higher among men compared to women (11.67% men vs. 9.95% women). Conversely, the percentage of women who have never smoked (65.19%) is significantly greater than that of men (28.33%). However, regarding former smokers, it appears that a higher proportion of older men ceased smoking compared to women (60% men vs. 24.86% women);
- **Alcohol consumption.** Regarding alcohol consumption patterns within the surveyed population, our data reveals distinct categories as presented in Figure A11. Consequently, our analysis demonstrates that 35.95% of the population refrains from alcohol consumption, while 43.48% of individuals consume alcohol occasionally, typically between 1 and 4 times per month. When segregating these data by gender, it becomes apparent that alcohol consumption and drinking frequency are more prevalent among men. Specifically, a higher proportion of men engage in alcohol consumption compared to women. In fact, nearly half of the female respondents (48.5%) reported abstaining from alcohol consumption in the past year;
- **Nutrition.** The simplified nutritional appetite questionnaire (SNAQ65) was the instrument used to assess the nutritional status and the risk of weight loss in older people. In the assessment using these instruments, it was determined that 90 individuals, constituting 37.2% of the population, were categorised as being at risk of malnutrition, as indicated by scores ranging from one to six. Conversely, 152 individuals (62.8% of the population) were identified as having no risk of malnutrition, possessing a SNAQ score of zero. This finding underscores that a considerable proportion of the population is susceptible to malnutrition. When it comes to women and men, the percentage or women at risk is 40.33% whereas in men the percentage is decreased (27.86%). Comparing the average for the SNAQ65 scores between women and men, no significant differences were found (t = 1.836; *p*-value = 0.0676);
- **Physical activity.** In the realm of the frequency of which participants do physical activity, our data unveil diverse engagement levels within the surveyed population (Figure A12). A significant portion of the population, comprising more than half, is characterised as sedentary, reflecting limited or negligible engagement in physical activity. Upon gender-based comparison of these data, the proportions exhibit similarity between men and women, with a marginal increment noted in men’s physical activity levels. Specifically, the percentage of women who rarely or never engage in exercise is slightly higher than that of men (62.98% women vs. 54.10% men), while the proportions of women engaging in physical activity 1–3 times a month (3.31%), or once a week (5.52%), are lower in comparison to men (6.56% and 11.48%, respectively).

### 3.4. Use of Health Resources

- The assessment of the **risk associated with medication use** was graded on a scale ranging from zero to eight. This scale revealed that 58.1% of the cohort were classified as being at low risk of inappropriate medication use (scoring between zero and three), while 101 individuals (41.7% of the cohort) were deemed to be at risk (scoring between four and eight). Whereas the percentage of women at risk represents 61.36% of the population, values for men are decreased, with less than half of the population (49.18%) detected to be at risk. No significant differences were found when comparing the adapted MRQ-10 score between women (MQR score = 3.217) and men (MQR score = 3.393) (t = 0.7959; *p*-value = 0.4269);
- **Number of visits to the doctor in the preceding year**. Most of the participants, comprising 93.75%, visited a doctor within the last year. On average, these individuals visited the doctor approximately 4.45 times per year. Only a minority (6.25%) did not have any doctor visits in the preceding 12 months;
- **Number of hospital admissions**. A total of 13.3% of the participants were hospitalised during the previous year. On average, these hospitalisations spanned a duration of 6.4 days per admission.

Differences between women and men data were not significant, regarding neither the visits to the doctor (93.89% of women visited the doctor with an average of 4.6 visits last year vs. 93.33% of men with an average of 4 visits; t = 0.9807; *p*-value = 0.3278) nor regarding the hospital admissions (11.67% of women were hospitalised with an average stay of 6.09 days vs. the 18.33% of men with an average of 7 days; t = 0.4720; *p*-value = 0.6404).

## 4. Discussion

The results provide an overview of the study sample in terms of health, well-being, quality of life, lifestyles and consumption of health resources. Compared with the results from the Valencian community health survey 2022 [29], where 78% of the adult population rated their health positively, our data show slightly lower scores for the general health status. This discrepancy is particularly notable among those over 65 years of age. Regarding **mental health** and according to the ESCV 2022 [29], which used the GHQ-12 questionnaire to assess mental health, the prevalence of individuals at risk for poor mental health is higher among women; however, our data showed that mental health outcomes are significantly better among men in our sample (PROMIS Mental Health T score of 47.9 for men compared to 43.76 for women). This survey also reveals a negative trend over time, with an increasing risk of poor mental health, particularly among men, which can explain our results. When it came to **anxiety and depression**, 24% of individuals in the referenced study indicated having issues [30], compared to 43.8% in our study who reported problems related to these conditions. Regards unwanted **loneliness,** our findings indicate that the majority of the population aged 65 and older did not experience feelings of loneliness (88%); this result contrasts sharply with data published in 2013 [31] which reported that 63.2% of individuals felt lonely. It is important to consider the time elapsed between the two studies, during which there have been increased opportunities for leisure and social interaction among older adults. Notwithstanding, our data align with the above study in terms of gender differences in loneliness. In our study, women were found to experience loneliness at a rate four to five times higher than men, while the earlier study reported that women were three times more likely to feel lonely compared to men. Concerning **mobility**, 60% of participants in the referenced study [30] reported some level of difficulty, whereas this figure was reduced to 27.2% in the elderly population of Valencia. Similarly, for **selfcare**, the difference is notable but less pronounced; A total of 24% of the study’s participants reported issues in [30], compared to 12.4% in our study. Furthermore, 24% of the participants in the mentioned study reported problems related to **pain/discomfort** [30], mirroring the trend observed in our findings. However, in our case, a significant proportion of the population (64.88%) experienced mild, moderate, severe, or extreme pain. Regarding **daily activities**, 48% of older individuals reported at least some difficulties [30], whereas only 23.4% of our participants faced challenges in performing these activities. The total Tilburg **frailty** indicator (TFI) for older individuals in the city of Valencia was 5.62. This is slightly higher than the TFI of 4.64 reported in the Zhang et al. study [32], which compares frailty data across different countries, indicating that the older population in Valencia exhibits less frailty compared to the broader Spanish population. When disaggregated into domains, the physical frailty indicator in our study was 2.90 for the older population in Valencia, lower than the 3.0 reported for the Spanish population in the mentioned study. However, in the psychosocial domain, our TFI was 1.50 for Valencia, compared to 1.18 for the Spanish population. Lastly, the TFI for the social domain was 1.9 for older individuals in Valencia, almost one point higher than the 1.01 reported for the Spanish population. These findings suggest that the overall lower frailty observed in Valencia’s older population is primarily attributable to better physical frailty outcomes. In contrast, the psychosocial and social frailty indicators are higher, indicating that the older population in Valencia is more affected in these domains compared to the broader Spanish population. In terms of the **prevalence of comorbidities**, it is evident that these increase with age, making it uncommon to find individuals aged 65 and older without any comorbid conditions. Our results corroborate with the ESCV 2022 survey [29], noting that comorbidities are more prevalent in women than in men. It also highlights a rise in major metabolic cardiovascular risk factors, including hypertension, high cholesterol, and diabetes. Hypertension and high cholesterol were among the most frequently reported comorbidities in our study, alongside leg and back pain. Finally, our results are also in line with the ESCV survey [29] on **dependency** levels that reveals that severe limitations affect 3.8% of the adult population. Additionally, the survey highlights that gender differences in limitations narrow with age, primarily due to a greater reduction in limitations among women over 64 years old. This trend might explain why, in our study of Valencia’s elderly population, the data are very similar for both sexes, with no significant differences observed.

Analysing lifestyles and comparing our results on **ICMs** with data from the National Statistics Institute (NSI), the proportion of individuals with normal weight is similar (42.52%). However, the percentage of underweight individuals in the NSI data is significantly lower at 2.58%. This discrepancy may be attributed to the fact that the NSI data encompasses the body mass index (BMI) for all adults in the Valencian community over the age of 18, rather than focusing solely on the elderly population. Furthermore, our data indicated that 23.55% of the participants were categorised as overweight, and 17.12% were identified as obese. Consequently, less than half of the surveyed population exhibits a normal weight status, while nearly 40% are characterised as overweight or obese. These findings are consistent with those reported by the NSI. Gender comparisons revealed that overweight and obesity are more prevalent among men, with a BMI significantly higher among them. This trend was observed in both the elderly population of Valencia and the NSI data for the general adult population. Regarding **tobacco consumption**, the ESCV 2022 [29] showed the same trend observed in our study, where the largest proportion of individuals are non-smokers. Considering gender, our results are in line with the data presented in the Valencian community health survey for tobacco and **alcohol** consumption [29] which stated the following: slightly higher current smoker prevalence among men, higher percentage of women who have never smoked than men, drinking frequency are higher among men, and almost half of the female respondents reported not drinking alcohol at all in the past year. Considering **nutritional assessment** and despite the lack of comparative data from other studies, ESCV 2022 [29] reinforced our findings to the extent of the survey that indicates that approximately 75% of the adult population in the Valencian community demonstrated moderate adherence to the Mediterranean diet, while about 15% reported consistently following it. Consequently, roughly 8% of adults with low adherence to the Mediterranean diet may correspond to the 37% of the older population identified in our data as being at risk of malnutrition. This correlation requires further investigation. Finally, in terms of **physical activity**, our findings about the sedentarism of the population studied are corroborated by the ESCV 2022 report [29], which estimates that nearly one-quarter of the adult population fails to meet WHO recommendations for physical activity [33].

Regarding the use of resources, the ESCV 2022 report [29] indicated that 86.3% of the population over the age of 64 consumes prescribed **medications**. Additionally, although to a lesser extent, self-medication is also prevalent in this population group. Consequently, the risk of medication misuse among older individuals is not unexpected. However, a detailed analysis of the underlying reasons for this medication misuse would be of significant interest. Concerning **medical consultations**, the ESCV 2022 report supports our findings by noting that the most frequently used health service was family medicine [29]. Regarding **hospital admissions**, the ESCV 2022 data indicates a decline in hospital admissions among the adult population in recent years; no comparison can be conducted as our results do not present data for that period of time.

## 5. Conclusions

This paper presents an overview of the health status of older people with frailty in the city of Valencia before implementing the ValueCare model. The results showed relevant data for the adaptation of the model to the Valencia community, considering the characteristics of older people in the city. In summary, older people in Valencia reported the following:

- Good health, considering physical and mental health, with differences among gender (men reported better physical and mental health than women) with sedentary lives and high levels of BMI;
- Good quality of life, better reported by men than by women;
- Healthy habits related to tobacco and alcohol consumption, and no risk of malnutrition;
- No big mobility problems or pain, although men reported lower levels of pain than women;
- Independent to perform selfcare and to perform their daily activities;
- No anxiety/depression (majority of the sample), but the prevalence of these symptoms among women were significantly higher than for men;
- Not frail (majority of the sample), but women presented significantly increased levels of social and physical frailty than men and higher levels of fear of falling;
- With absence of loneliness, with difference among genders, being women who reported higher scores;
- Living with comorbidities.

Given the discrepancies found between our results and those reported in some of the previous studies analysed, it would be interesting to further compare our findings with additional studies. Investigating the underlying reasons for the highlighted differences would provide valuable insights and enhance the reliability of our findings.

## Data Availability

The original contributions presented in this study are included in the article. Further inquiries can be directed to the corresponding author(s).

## Appendix A

**Table A1:** Inclusion criteria.

Criteria	Questionnaire	Inclusion Criteria
Age	-	≥60 years
Frailty	FRAIL scale [14]	FRAIL scale score ≥ 1
Dependency	Barthel index [15]	Barthel index score ≥ 60
Cognitive impairment	Pfeiffer SMPQ scale [16]	Pfeiffer SMPQ scale score between 0–2

**Table A2:** Outcome measures used in the comprehensive evaluation of health.

Outcome	Measure	Instrument	Reliability and validity
Health, well-being, and quality of life	Physical and Mental HR-QoL	PROMIS-10	Cella et al., 2010 [34]
	HR-QoL	EQ5D5L	Hermand et al., 2011 [20]
	Frailty	Tilburg frailty indicator	Fürstenau, D. et al. 2022 [35]
	Comorbidities	ICHOM older person set	Not applicable
	Loneliness	UCLA 3-item loneliness scale	Trucharte et al., 2023 [36]
	Activities of daily live	Modified 10-item Barthel index	González et al., 2018 [37]
	Falls	Visual analogue scale for fear of falling	Scheffer et al., 2010 [38]
Lifestyle behaviour	BMI	ICHOM older person set	Not applicable
	Smoking status	ICHOM older person set	Not applicable
	Alcohol consumption	ICHOM older person set	Not applicable
	Physical activity	One item of the SHARE-Frailty One item of the international physical activity questionnaire (IPAQ)	Not applicable
	Nutrition	SNAQ65 +	Wijnhoven et al., 2011 [26]
	Medication intake	Medication risk questionnaire (MRQ-10)	Not applicable
Care use	Healthcare utilisation	Modified SMRC healthcare utilisation questionnaire	Not applicable

**Table A3:** Sociodemographic data.

Living situation	lived alone in their own residence	45.83%
	lived with a partner, family, or friends	53.3%
	Lived in a residence	0.42%
	Lived with a helper	0.42%
Marital status	Married	47.5%
	Widowed	31.67%
	Divorced	12.92%
	Single	7.5%
	Long-term cohabitation	0.42%
Level of education	No education	8.3%
	Primary education	35%
	Lower secondary education	12.08%
	Upper secondary education	15%
	Post-secondary non-tertiary education	5.42%
	Tertiary education	6.67%
	Bachelor’s	15.83%
	Master’s	0.83%
	Doctorate	0.83%
